# Current situation and associated factors of clinical narrative competence of nurses in selected hospitals in Guangxi, China: a cross-sectional study

**DOI:** 10.3389/fmed.2026.1898040

**Published:** 2026-08-14

**Authors:** Senlan Zhang, Kaiyong Huang, Liuyi Chen, Yingming Liao, Huabin Su, Jinmeng Huang

**Affiliations:** 1 Department Two of Gynecology, The First Affiliated Hospital of Guangxi Medical University, Nanning, Guangxi, China; 2 Department of Occupational Health and Environmental Health, School of Public Health, Guangxi Medical University, Nanning, Guangxi, China; 3 China (Guangxi)-ASEAN Engineering Research Center of Big Data for Public Health, Guangxi Medical University, Nanning, China; 4 University Engineering Research Center of China (Guangxi)-ASEAN Intelligent Health Emergency Response, Nanning, Guangxi, China; 5 School of Humanities and Social Sciences, Guangxi Medical University, Nanning, Guangxi, China; 6 School of Information and Management, Guangxi Medical University, Nanning, Guangxi, China; 7 Ministry of Science and Education, Jiangbin Hospital of Guangxi Zhuang Autonomous Region, Nanning, Guangxi, China; 8 Educational Evaluation and Faculty Development Center, Guangxi Medical University, Nanning, Guangxi, China

**Keywords:** associated factors, clinical narrative competence, cross-sectional study, narrative medicine, nurse

## Abstract

**Background:**

Patient-centered care, which emphasizes clinical narrative competence of nurses, is receiving increasing attention. However, there is limited evidence indicating the level of clinical narrative competence of nurses. This study aimed to explore the current situation and associated factors of the clinical narrative competence of nurses in selected hospitals in Guangxi, China.

**Methods:**

This cross-sectional study involved 316 nurses from seven affiliated hospitals of Guangxi Medical University and Guangxi University of Chinese Medicine. A self-administered questionnaire was used for data collection through an online survey platform. T-tests and one-way ANOVA were utilized to determine significant differences for the variables. Multiple linear regression analysis was employed to explore the significant variables associated with the clinical narrative competence of nurses.

**Results:**

Univariate analysis showed that everal factors were significantly associated with clinical narrative competence (*p* < 0.003 for all), including: “the level of knowledge about narrative medicine”; “believing that it’s necessary to understand the stories and feelings of patients or their families”; “believing that listening to patients’ accounts helps medical staff understand patients more deeply, thereby prompting them to provide more personalized and humanized treatment to patients”; “willing to continue or plan to study narrative medicine”; and “believing that it’s necessary to develop narrative medicine research and practice in clinical settings.” Multiple linear regression analysis revealed that “believing that it is necessary to develop narrative medicine research and practice in clinical settings” (*p* = 0.002), “believing that listening to patients’ accounts helps medical staff understand patients more deeply” (*p* = 0.009), and “the level of knowledge about narrative medicine” (*p* = 0.011) were significant associated factors of the clinical narrative competence of nurses.

**Conclusion:**

Attitudes towards narrative medicine were significantly associated with the clinical narrative competence of nurses. It is suggested that the knowledge and skills related to narrative medicine and narrative nursing should be incorporated into the core competence development of nurses. Moreover, it is necessary to gradually introduce narrative nursing practice into clinical nursing. These measures may enhance nurses’ clinical narrative competence and provide high-quality nursing services to patients.

## Introduction

Narrative medicine was initially conceptualized defined as practicing medicine with narrative competence, highlighting the critical interaction between science and humanities by Rita Charon in 2001 (1). It is regarded as an important tool for obtaining, understanding and integrating all aspects of various participants in a specific disease experience, which offers a structured framework to enhance medical staff’s responses to patient narratives, guiding the development of related interventions and training (2, 3). Narrative medicine can help healthcare professionals to understand patients’ experiences with diseases, empathize with them, and meet their narrative needs, thereby discovering the essence of problems faced and providing higher-quality medical and nursing services. Nurses play an extremely important role in the provision of medical services, emphasizing a blend of scientific rigor and humanistic care (4). Therefore, in recent years, narrative medicine has become a significant focus of research within the nursing field.

Over the past two decades, the nurse–patient relationship in clinical practice has shifted from the traditional disease-centered model to a patient-centered one. While paying attention to the development of medical technology, patients also have a strong demand for emotional, psychological, social and information support, and their requirements are increasing. This places very high demands on the level of nurses’ clinical narrative competence (5, 6). However, the success of narrative medicine depends on the clinical narrative competence of medical staff, especially nurses (5). Therefore, enhancing the clinical narrative competence of nurses is key to ensuring comprehensive nursing and improving the effectiveness of medical services.

Clinical narrative competence, which refers to the ability to pay attention to, understand, and promptly respond to problems from the patient’s perspective while seeking solutions, has emerged as a key concept in nursing practice (7). It is centered on empathy and reflection, which involves listening and accepting, digesting and absorbing, analyzing and interpreting, and responding to the stories and predicaments of patients. This approach helps healthcare professionals to enhance their empathy towards patients, their affinity with them, and their self-reflection on their own behaviors during medical activities. Clinical narrative competence is of vital importance in ensuring that patients receive the best, most humane and high-quality medical and nursing services, and represents an innovative approach to improving patients’ health conditions (4, 8). A previous study found that patients and their caregivers have a strong need to be heard. They are willing to disclose their understanding of the disease and their psychological conditions to doctors and nurses, so that the medical staff can better understand the patients’ experiences, thereby maintaining smooth and friendly communication with medical staff and promoting a good treatment outcome (9). Some studies revealed that clinical narrative competence is beneficial in enhancing nurses’ humanistic care ability, empathy and affinity, promoting self-reflection, and improving their nursing practices (3, 5, 10). Furthermore, studies have highlighted the positive impact of the clinical narrative competence of nurses on improving communication skills and reducing negative emotions in patients (11).

Over the past decade, research on narrative medicine in China has significantly increased. Some universities have already incorporated clinical narrative competence training into medical education, or are preparing to do so, but the implementation of narrative medicine in clinical nursing remains limited (5, 12). There is little evidence indicating the level of clinical narrative competence of nurses in China. Therefore, an in-depth research is required to explore the main factors associated with the clinical narrative competence of nurses so as to provide necessary suggestions for formulating targeted intervention measures. This study comprehensively examined the current situation and the association between narrative medicine knowledge, attitudes, and clinical narrative competence, while most existing studies in China only focus on the level of narrative competence itself. The study focused on nurses in Guangxi, a southwestern region of China, where narrative medicine research is still limited, providing regional-specific evidence for the development of narrative medicine in nursing.

## Methods

### Study participants and procedures

This study was conducted at seven affiliated hospitals of two major medical universities in Nanning, Guangxi province, China. These included the First Affiliated Hospital of Guangxi Medical University (1^st^AFGXMU), the Second Affiliated Hospital of Guangxi Medical University (2^nd^AFGXMU), the Affiliated Cancer Hospital of Guangxi Medical University (ACHGXMU), the Affiliated Stomatology Hospital of Guangxi Medical University (ASHGXMU), the Wuming Hospital Affiliated to Guangxi Medical University (WHAGXMU), the First Affiliated Hospital of Guangxi University of Chinese Medicine (1^st^AFGXUCM), and the Ruikang Hospital Affiliated to Guangxi University of Chinese Medicine (RHAGXUCM). The target population was full-time nurses. Participants were recruited using convenience sampling. Inclusion criteria were full-time nurses, willing to participate in this survey, and competent in providing informed consent. Exclusion criteria were part-time nurses, stopping the survey halfway, providing incomplete or invalid responses to the questionnaire. According to the sample size estimation principle for multivariable studies, the sample size is generally set at 10–20 times the number of independent variables. In this study, there were 16 independent variables. Considering the possibility of invalid questionnaires during the survey, the sample size was increased by 20%, resulting in a required sample of 192–384 participants.

This cross-sectional study was administered from February 2024 to July 2024 at the seven affiliated hospitals. Data collection was via an online questionnaire, which was uploaded to a professional online survey platform named “Wen Juan Xing.” The questionnaire provided a comprehensive overview of the study’s objectives, research methodology, and the requirement for informed consent. The online questionnaire’s link was sent to the head nurses of various nursing departments in the hospitals, and then forwarded to front line nurses. A response to all questions was required, and each IP address could only be submitted once. Before the survey, all participants were informed that their participation was voluntary, anonymous, and confidential. Initially, a small-scale preliminary survey involving 20 nurses was conducted at 1^st^AFGXMU using “Wen Juan Xing.” Based on the results of this preliminary survey, a portion of the questionnaire was revised by three experts in epidemiology and statistics. Finally, the question of whether the respondent had participated in nursing teaching work was added to the questionnaire, while annual income was deleted because most participants were reluctant to disclose this information.

### Sociodemographic characteristics questionnaire

The sociodemographic characteristics included: sex, age, ethnicity (Han, Zhuang, and others); marital status (unmarried, married, divorced); educational background (junior college, bachelor’s degree, master’s degree and above); professional title (junior, intermediate, associate senior and above); position (head nurse, front line nurse); work experience; hospital; and whether they had participated in nursing teaching work.

### Attitudes towards narrative medicine questionnaire

The attitudes towards narrative medicine questionnaire included six self-designed items. The item development was guided by Rita Charon’s classic narrative medicine theoretical framework (1). Combined with domestic published cross-sectional studies on nurses and college students’ narrative cognition (2, 3), we extracted six core latent dimensions: basic knowledge cognition, clinical value recognition, empathy cognition, listening value recognition, learning willingness, and clinical implementation recognition. Original draft of 12 candidate questions was first compiled covering the six dimensions; three experts in epidemiology and statistics screened redundant, ambiguous items, merging similar descriptions and deleting repetitive content, and finally retained six representative single items corresponding to six independent dimensions without overlap. Item I. “The level of knowledge about narrative medicine” (1 = I’ve never heard about it, 2 = I know a little bit about it, 3 = I know well about it). Item II. “agreeing that clinical nursing work should be patient-centered and enhance patient engagement” (1 = Yes, 2 = No). Item III. “believing that it is necessary to understand the stories and feelings of patients or their families” (1 = Yes, 2 = No). Item IV. “believing that listening to patients’ accounts helps medical staff understand patients more deeply, thereby prompting them to provide more personalized and humanized treatment to patients” (1 = Yes, 2 = No). Item V. “willing to continue or plan to study narrative medicine” (1 = Yes, 2 = No). Item VI. “believing that it is necessary to develop narrative medicine research and practice in clinical settings” (1 = Yes, 2 = No).

### Narrative competence scale

The narrative competence scale was developed by Wanzhen Ma (13) through the integration of narrative theory, story theory and Noddings’ care theory. The scale serves as a tool for evaluating the medical narrative competence of medical staff and healthcare professionals and has been widely used in the public domain (3). It consists of three dimensions and a total of 27 items. The three dimensions include: attention and listening (9 items); understanding and responding (12 items); and reflection and representation (6 items) (3, 13). Responses for each item are recorded on a 5-point Likert scale. The total score for narrative competence ranges from 27 to 135, with higher scores indicating greater medical narrative skills among nurses. In this study, the Cronbach’s *α* coefficient of the scale is 0.970, indicating good reliability.

### Statistical analysis

Statistical analysis was conducted using IBM SPSS version 22.0 (IBM Corp., Armonk, NY, United States). Categorical variables are summarized using frequencies and percentages, whereas quantitative data are presented as mean ± standard deviation (SD). Based on the optimal model, t-tests and one-way ANOVA were used to determine whether there was a significant difference between the means of the variables. To mitigate multicollinearity, 16 separate tests explored the relationships between sociodemographic characteristics and attitudes towards narrative medicine and narrative competence score. To control for type I errors from multiple testing, a Bonferroni correction was applied, setting the threshold for significance at *p*-values of < 0.003 (0.05/16). A multiple linear regression model was used to select the significant variables associated with narrative competence, controlling for gender, age, educational background, professional title, and hospital. The tolerance values and variance inflation factors (VIF) were calculated to assess multicollinearity, with the tolerance values < 0.1 or the VIF > 10 indicating severe collinearity. *p* < 0.05 was considered statistically significant.

## Results

### Demographic characteristics of the study sample

A total of 330 questionnaires were distributed and 322 were collected, including 316 valid questionnaires, with an effective rate of 95.7%. The remaining 6 questionnaires were eliminated for the following reasons: four questionnaires were incomplete or invalid, and two were logically contradictory. A comprehensive overview of the demographic characteristics and attitudes towards narrative medicine is provided in Table 1. Among the participants, 268 (84.8%) were female, 218 (69.0%) were from the Han ethnicity, most of whom 68.4%) were aged ≤ 35 years old, and 231 (73.1%) were married. The majority of nurses (77.2%) held a bachelor’s degree, 159 (50.3%) had an intermediate professional title, and most (92.7%) worked as front line nurses, followed by 7.3% as head nurses. Of the 316 participants, 73 (23.1%) were from the 1^st^AFGXMU, over a third (32.0%) had been engaged in nursing work for 6–10 years, and 284 (89.9%) had participated in nursing teaching work. In terms of the level of knowledge about narrative medicine, 205 (64.9%) had never heard about it, but 284 (89.9%) agreed that clinical nursing work should be patient-centered and enhance patient engagement. More than 90.0% of the respondents thought that it was necessary to understand the stories and feelings of patients or their families, and approximately 90.0% of the nurses believed that listening to patients’ accounts helps medical staff understand patients more deeply, thereby prompting them to provide patients with a more personalized and humanized treatment. More than 74.0% of the participants responded positively to the statements “willing to continue or plan to study narrative medicine” and “it is necessary to develop narrative medicine research and practice in clinical settings” (Table 1).

**Table 1:** Sociodemographic characteristics and attitudes towards narrative medicine of participants.

Variable	N	%
Gender		
Male	48	15.2
Female	268	84.8
Age		
≤ 30 years old	115	36.4
31–35 years old	101	32.0
36–40 years old	64	20.2
≥ 41 years old	36	11.4
Ethnicity		
Han ethnicity	218	69.0
Zhuang ethnicity	87	27.5
Others	11	3.5
Marital status		
Unmarried	82	25.9
Married	231	73.1
Divorced	3	1.0
Educational background		
Junior college	56	17.7
Bachelor’s degree	244	77.2
Master’s degree and above	16	5.1
Professional title		
Junior	124	39.2
Intermediate	159	50.3
Associate senior and above	33	10.4
Position		
Head nurses	23	7.3
Front line nurses	293	92.7
Work of experience		
≤ 5 years	84	26.6
6–10 years	101	32.0
11–15 years	73	23.1
≥ 16 years	58	18.3
Whether to participate in nursing teaching work?		
Yes	284	89.9
No	32	10.1
Hospital		
1^st^AFGXMU	73	23.1
2^nd^AFGXMU	56	17.7
ACHGXMU	35	11.1
ASHGXMU	48	15.2
WHAGXMU	23	7.3
1^st^AFGXUCM	47	14.9
RHAGXUCM	34	10.7
The level of knowledge about narrative medicine		
I’ve never heard about it	205	64.9
I know a little bit about it	80	25.3
I know well about it	31	9.8
Agreeing that clinical nursing work should be patient-centered and enhance patient engagement.		
No	32	10.1
Yes	284	89.9
Believing that it’s necessary to understand the stories and feelings of patients or their families.		
No	16	5.1
Yes	300	94.9
Believing that listening to patients’ accounts helps medical staff understand patients more deeply, thereby prompting them to provide more personalized and humanized treatment to patients.		
No	32	10.1
Yes	284	89.9
Willing to continue or plan to study narrative medicine.		
No	80	25.3
Yes	236	74.7
Believing that it’s necessary to develop narrative medicine research and practice in clinical settings.		
No	68	21.5
Yes	248	78.5

1^st^AFGXMU, The First Affiliated Hospital of Guangxi Medical University; 2^nd^AFGXMU, The Second Affiliated Hospital of Guangxi Medical University; ACHGXMU, The Affiliated Cancer Hospital of Guangxi Medical University; ASHGXMU, The Affiliated Stomatology Hospital of Guangxi Medical University; WHAGXMU, Wuming Hospital Affiliated to Guangxi Medical University; 1^st^AFGXUCM, The First Affiliated Hospital of Guangxi University of Chinese Medicine; RHAGXUCM, Ruikang Hospital Affiliated to Guangxi University of Chinese Medicine.

### Scores of clinical narrative competence in nurses

As shown in Table 2, the total scores of clinical narrative competence in nurses was 108.00 ± 16.12. The scores of attention and listening, understanding and responding, and reflection and representation were 35.07 ± 5.11, 48.63 ± 7.80, 24.31 ± 3.83, respectively.

**Table 2:** Score of clinical narrative competence in nurses.

Variables	Items	Total score	Mean ± SD
Clinical narrative competence	27	27 ~ 135	108.00 ± 16.12
Attention and listening	9	9 ~ 45	35.07 ± 5.11
understanding and responding	12	12 ~ 60	48.63 ± 7.80
Reflection and representation	6	6 ~ 30	24.31 ± 3.83

### Univariate analysis of clinical narrative competence

As shown in Table 3, several factors were significantly associated with clinical narrative competence (*p* < 0.003 for all), including: “the level of knowledge about narrative medicine”; “believing that it’s necessary to understand the stories and feelings of patients or their families”; “believing that listening to patients’ accounts helps medical staff understand patients more deeply, thereby prompting them to provide more personalized and humanized treatment to patients”; “willing to continue or plan to study narrative medicine”; and “believing that it’s necessary to develop narrative medicine research and practice in clinical settings.”

**Table 3:** Univariate analysis on associated factors of narrative competence.

Variable	Mean ± SD	*p-*value
Gender		0.777
Male	107.39 ± 20.57	
Female	108.11 ± 15.23	
Age		0.234
≤ 30 years old	109.60 ± 15.57	
31–35 years old	106.10 ± 16.68	
36–40 years old	106.48 ± 14.98	
≥ 41 years old	110.92 ± 17.82	
Ethnicity		0.550
Han ethnicity	107.72 ± 16.09	
Zhuang ethnicity	109.21 ± 15.98	
Others	104.09 ± 18.26	
Marital status		0.127
Unmarried	110.61 ± 14.68	
Married	106.96 ± 16.45	
Divorced	117.33 ± 22.81	
Educational background		0.123
Junior college	109.04 ± 15.59	
Bachelor’s degree	108.29 ± 16.43	
Master’s degree and above	100.06 ± 10.80	
Professional title		0.662
Junior	108.92 ± 16.13	
Intermediate	107.63 ± 14.70	
Associate senior and above	106.36 ± 22.00	
Position		0.320
Head nurses	104.78 ± 22.71	
Front line nurses	108.25 ± 15.51	
Work of experience		0.998
≤ 5 years	107.80 ± 16.01	
6–10 years	107.93 ± 17.22	
11–15 years	108.29 ± 14.00	
≥ 16 years	108.07 ± 17.16	
Whether to participate in nursing teaching work?		0.525
Yes	108.20 ± 16.11	
No	106.28 ± 16.29	
Hospital		0.085
1^st^AFGXMU	106.40 ± 18.55	
2^nd^AFGXMU	107.79 ± 12.18	
ACHGXMU	111.29 ± 13.33	
ASHGXMU	110.98 ± 14.30	
WHAGXMU	114.39 ± 14.93	
1^st^AFGXUCM	104.51 ± 16.11	
RHAGXUCM	104.74 ± 20.24	
The level of knowledge about narrative medicine		0.001
I’ve never heard about it	106.35 ± 16.14	
I know a little bit about it	108.40 ± 16.19	
I know well about it	117.90 ± 12.08	
Agreeing that clinical nursing work should be patient-centered and enhance patient engagement.		0.006
No	100.62 ± 25.88	
Yes	108.84 ± 14.45	
Believing that it’s necessary to understand the stories and feelings of patients or their families.		<0.001
No	93.19 ± 24.58	
Yes	108.79 ± 15.20	
Believing that listening to patients’ accounts helps medical staff understand patients more deeply, thereby prompting them to provide more personalized and humanized treatment to patients.		<0.001
No	97.03 ± 20.89	
Yes	109.24 ± 15.04	
Willing to continue or plan to study narrative medicine		<0.001
No	102.51 ± 16.37	
Yes	109.86 ± 15.63	
Believing that it’s necessary to develop narrative medicine research and practice in clinical settings.		<0.001
No	99.60 ± 15.59	
Yes	110.31 ± 15.51	

SD, Standard Deviation; 1^st^AFGXMU, The First Affiliated Hospital of Guangxi Medical University; 2^nd^AFGXMU, The Second Affiliated Hospital of Guangxi Medical University; ACHGXMU, The Affiliated Cancer Hospital of Guangxi Medical University; ASHGXMU, The Affiliated Stomatology Hospital of Guangxi Medical University; WHAGXMU, Wuming Hospital Affiliated to Guangxi Medical University; 1^st^AFGXUCM, The First Affiliated Hospital of Guangxi University of Chinese Medicine; RHAGXUCM, Ruikang Hospital Affiliated to Guangxi University of Chinese Medicine.

### Multiple linear regression analysis of factors associated with clinical narrative competence

To further explore the important factors associated with clinical narrative competence, a multiple linear regression model was conducted. The collinearity diagnosis of the final regression model shows that the VIF of all variables ranged from 1.040 to 2.368, and the tolerance values ranged from 0.422 to 0.961, indicating no multicollinearity among these variables (Supplementary Table S1). Table 4 shows that “believing that it is necessary to develop narrative medicine research and practice in clinical settings” (*p* = 0.001), “believing that listening to patients’ accounts helps medical staff understand patients more deeply, thereby prompting them to provide more personalized and humanized treatment to patients” (*p* = 0.010), and “the level of knowledge about narrative medicine” (*p* = 0.012) were significant associated factors of clinical narrative competence of nurses.

**Table 4:** Multiple linear regression analysis results for associated factors of narrative competence.

Variables	Unstandardized coefficient		Standardized coefficient	T	*p-*value	95% CI
	B	Standard errors	Beta			
Believing that it’s necessary to develop narrative medicine research and practice in clinical settings.	3.267	1.024	0.212	3.195	0.002	1.142–5.392
Believing that listening to patients’ accounts helps medical staff understand patients more deeply, thereby prompting them to provide more personalized and humanized treatment to patients.	2.415	0.931	0.150	2.597	0.009	1.841–4.349
The level of knowledge about narrative medicine.	2.108	0.827	0.141	2.556	0.011	1.360–3.627

CI, Confidence interval. Model was controlled for gender, age, educational background, professional title, and hospital. Model-level statistics: *F* = 14.935, *p* < 0.001, *R*^2^ = 0.704, adjusted *R*^2^ = 0.697.

## Discussion

This study examined the current state and associated factors of the clinical narrative competence among nurses in southern China and identified “the level of knowledge about narrative medicine” as a key associated factor. The mean scores of clinical narrative competence for “I know quite a lot about narrative medicine” was the highest, which means that those nurses had the best clinical narrative competence. A similar study has also indicated that the limited formal education on narrative medicine concepts and insufficient training in therapeutic communication skills are identified as the main obstacles to clinical narrative competence (2). At present, the implementation of narrative medicine in clinical nursing is still limited in China, with the majority of hospitals and medical colleges failing to offer courses and training on the subject. Nurses mainly learned about narrative medicine through various information sources such as the Internet, academic papers, lectures, and conferences, etc. One study reported that the participants who attended the narrative medicine seminar demonstrated an improvement in their knowledge on the subject during the subsequent test and later applied these narrative skills in teaching and clinical work (14). Similarly, Ross et al. found that after participants completed the narrative medicine course, their clinical narrative competence was significantly improved compared to before the course (15). A study conducted in China indicated that medical staff expanded their narrative medicine knowledge and clinical narrative competence through involving themselves in the process of narrative medicine teaching and training (e.g., reading narrative medicine-related books and watching related movies), which can provide a better understanding of the patient’s needs and better support (16).

However, it is worth noting that although 64.9% of participants reported never having heard of narrative medicine, their overall clinical narrative competence scores were relatively high (mean ≈ 106 out of a possible 135). The “Narrative Competence Scale” (13) was developed by integrating narrative theory, story theory, and Noddings’ care theory, and its items certainly overlap with core nursing competencies like empathy, attentive listening, and patient-centered care. These are skills that are cultivated in nursing education and clinical experience independently of formal exposure to the specific concept of “narrative medicine.” Therefore, the relatively high scores likely reflect these acquired foundational humanistic skills. The lack of formal knowledge about narrative medicine as a distinct field does not negate the existence of these skills. However, this distinction underscores that while nurses may possess the raw materials for narrative practice, they lack the structured knowledge and training to fully harness and consciously apply these skills as “narrative competence” in a way that can be systematically refined and integrated into their clinical workflow. This supports the argument that formal narrative medicine teaching and training is still crucial to move from innate humanistic care to structured, expert narrative practice.

Narrative medicine teaching and training enable nurses to master the knowledge and skills of narrative medicine well, which helps to enhance their ability to empathize (4). Empathy is one of the core components of clinical narrative competence, which facilitates nurse–patient communication and exchange. Some studies found that the insufficiency of formal education and training in narrative medicine leads to most nurses having only a superficial understanding of narrative medicine (2). This is the main reason for the lack of narrative medicine knowledge and clinical narrative competence and is also a major barrier to implementing narrative practices (2, 17). Therefore, nursing managers and educators can incorporate narrative medicine into the teaching and training curriculum, enabling nurses to acquire more knowledge of narrative medicine, cultivating their ability to empathize and improving their clinical narrative competence.

This study also indicated that “believing that listening to patients’ accounts helps medical staff understand patients more deeply, thereby prompting them to provide more personalized and humanized treatment to patients” was another significant determinant of clinical narrative competence in nurses. Narrative medicine education highlights the cultivation of medical staff’s listening ability along with their skills of understanding and responding. The emphasis that narrative nursing places on attentive listening in patient communication aims to fully understand their conditions, identify the causes of negative emotions such as anxiety in patients, and to provide appropriate psychological guidance through empathy so as to promptly respond to their needs, thereby facilitating the smooth progress of treatment and nursing (18). Some studies have suggested that listening to patients’ accounts could increase healthcare providers’ understanding of the disease history and characteristics during patient encounters, improving disease diagnosis and treatment through empathy, trust, and partnership (16, 19). A qualitative study found that active listening contributed to increasing patients’ trust and encouraged patients to disclose more information, which in turn enables nurses to comprehend patient needs, develop individualized care plans based on patient preferences, and ultimately improve patient outcomes (2). Therefore, in order to enhance the clinical narrative competence of nurses, the nursing management department should offer training that focuses on the abilities and skills of active listening (2, 7). At the same time, we also suggest that narrative medicine as a course should be incorporated into the regular teaching curriculum of the nursing major, and that regular narrative medicine training is conducted in clinical settings.

Additionally, the present study also revealed that “believing that it is necessary to develop narrative medicine research and practice in clinical settings” was a significant associated factor of the clinical narrative competence of nurses. Many studies described that their narrative medicine interventions in the clinical practice of nurses are feasible, acceptable, and effective (20, 21). In several studies, nurses reported a positive benefit from narrative medicine practice in clinical nursing, which they characterized as an emotionally compelling experience that provided them with context and invited introspection to protect themselves against burnout (20–22). These measures may contribute to improving the working efficiency and clinical narrative competence of nurses, thereby improving the accuracy of diagnosis and treatment. Previous studies found that nurses described the narrative medicine intervention as beneficial to their well-being and reported increased empathy, self-awareness, quality of humanistic care, teamwork and resilience (23, 24). Available data indicate that implementing narrative medicine practices in clinical settings may reduce feelings of isolation by emphasizing shared experiences and emotions, and enhance the sense of community among medical staff, as well as increase empathy towards patients and their families, thereby improving the clinical narrative competence of nurses (25). However, challenges to implementing narrative medicine interventions in clinical settings may include clinician time limitations and scheduling conflicts resulting in participation attrition (25). Therefore, in order to enhance the clinical narrative competence of nurses, medical colleges and hospitals should actively conduct research on narrative medicine and explore the most effective strategies and specific methods for implementing narrative medicine practices in clinical work, which is particularly important.

Although the multiple linear regression analysis did not indicate that “believing that it is necessary to understand the stories and feelings of patients or their families,” and being “willing to continue or plan to study narrative medicine” were significant associated factors of the clinical narrative competence of nurses, the univariate analysis showed these factors were associated with higher clinical narrative competence scores. This might be due to the small sample size of the study. The two perspectives mentioned above are all positive aspects of the learning and practice of narrative medicine. A large number of studies have shown that participating in narrative medicine learning and training, providing patient-centered and humanized care, actively listening to the stories of patients, is significantly positively correlated with nurses’ clinical narrative competence (14, 15, 18, 19). In conclusion, the study’s findings highlight the significant associated factors of clinical narrative competence among nurses. Participating in narrative medicine learning and training may potentially enhance nurses’ clinical narrative competence. Future longitudinal or intervention studies are required to establish the causal effects of narrative medicine training on clinical narrative competence.

## Limitations

The present study also has several limitations. First, the cross-sectional design of the study limits the ability to establish causality between narrative medicine learning and training and the clinical narrative competence of nurses. Therefore, a longitudinal design study can be used in future studies. Additionally, all data were collected via self-reported questionnaires, which may introduce common recall bias and social desirability effects, potentially impacting the accuracy of the results. Consequently, a mixed-method approach can be adopted in future studies, which includes a quantitative survey and qualitative interviews. Third, the study sample was drawn from only one province in southwest China, potentially limiting the generalizability of the findings to other provinces in China. Therefore, it is extremely important to conduct a nationwide multi-center study with a larger sample to obtain more scientific and accurate results.

## Conclusion

The present study explored the current state and associated factors of the clinical narrative competence of nurses in southern China. These findings provide initial insights into the factors that significantly associated with the clinical narrative competence of nurses—namely “believing that it is necessary to develop narrative medicine research and practice in clinical settings,” “believing that listening to patients’ accounts helps medical staff understand patients more deeply,” and “the level of knowledge about narrative medicine.”

## Data Availability

The original contributions presented in the study are included in the article/Supplementary material, further inquiries can be directed to the corresponding author.
