# Supplementary material for: Current situation and associated factors of clinical narrative competence of nurses in selected hospitals in Guangxi, China: a cross-sectional study
Source: Front Med (Lausanne). 2026 Aug 14;13:1898040. doi: 10.3389/fmed.2026.1898040 (PMC13536471; doi:10.3389/fmed.2026.1898040)
Supplement: Supplementary file 1 [file Table_1.docx]

**Supplementary Table 1.** Variance inflation factor (VIF) and tolerance tests results.

| **Variable** | **Tolerance** | **VIF** |
| --- | --- | --- |
| Gender | 0.912 | 1.096 |
| Age | 0.422 | 2.368 |
| Educational background | 0.938 | 1.066 |
| Professional title | 0.428 | 2.336 |
| Hospital | 0.961 | 1.040 |
| The level of knowledge about narrative medicine | 0.904 | 1.105 |
| Agreeing that clinical nursing work should be patient-centered and enhance patient engagement. | 0.850 | 1.176 |
| Believing that it’s necessary to understand the stories and feelings of patients or their families. | 0.718 | 1.393 |
| Believing that listening to patients’ accounts helps medical staff understand patients more deeply, thereby prompting them to provide more personalized and humanized treatment to patients. | 0.729 | 1.372 |
| Willing to continue or plan to study narrative medicine. | 0.683 | 1.464 |
| Believing that it’s necessary to develop narrative medicine research and practice in clinical settings. | 0.633 | 1.580 |
